# Students’ attitudes, beliefs and perceptions surrounding 2SLGBTQIA + health education and inclusiveness in Canadian physiotherapy programs

**DOI:** 10.1186/s12889-023-16554-2

**Published:** 2023-08-30

**Authors:** Codie A. Primeau, Holly T. Philpott, Kyle Vader, Janelle Unger, Christina Y. Le, Trevor B. Birmingham, Joy C. MacDermid

**Affiliations:** 1 School of Physical Therapy, Faculty of Health Sciences, Western University, London, ON Canada; 2 Wolf Orthopaedic Biomechanics Laboratory, Fowler Kennedy Sport Medicine Clinic, Western University, London, ON Canada; 3 School of Health and Rehabilitation Sciences, Faculty of Health Sciences, Western University, London, ON Canada; 4 Bone and Joint Institute, Western University, London, ON Canada; 5 School of Rehabilitation Therapy, Queen’s University, Kingston, ON Canada; 6 Department of Physical Therapy, Faculty of Rehabilitation Medicine, University of Alberta, Edmonton, AB Canada

**Keywords:** Physiotherapy, Education, Inclusiveness, LGBTQ +, LGBTQ + health, Survey, EDI

## Abstract

**Background:**

Patients who identify as 2SLGBTQIA + report negative experiences with physiotherapy. The objectives were to evaluate student attitudes, beliefs and perceptions related to 2SLGBTQIA + health education and working with individuals who identify as 2SLGBTQIA + in entry-level physiotherapy programs in Canada and to evaluate physiotherapy program inclusiveness towards 2SLGBTQIA + persons.

**Methods:**

We completed a nationwide, cross-sectional survey of physiotherapy students from Canadian institutions. We recruited students via email and social media from August-December 2021. Frequency results are presented with percentages. Logistic regression models (odds ratios [OR], 95%CI) were used to evaluate associations between demographics and training hours with feelings of preparedness and perceived program 2SLGBTQIA + inclusiveness.

**Results:**

We obtained 150 survey responses (mean age = 25 years [range = 20 to 37]) from students where 35 (23%) self-identified as 2SLGBTQIA + . While most students (≥ 95%) showed positive attitudes towards working with 2SLGBTQIA + patients, only 20 students (13%) believed their physiotherapy program provided sufficient knowledge about 2SLGBTQIA + health and inclusiveness. Students believed more 2SLGBTQIA + training is needed (*n* = 137; 92%), believed training should be mandatory (*n* = 141; 94%) and were willing to engage in more training (*n* = 138; 92%). Around half believed their physiotherapy program (*n* = 80, 54%) and clinical placements (*n* = 75, 50%) were 2SLGBTQIA + -inclusive and their program instructors (*n* = 69, 46%) and clinical instructors (*n* = 47, 31%) used sex/gender-inclusive language. Discrimination towards 2SLGBTQIA + persons was witnessed 56 times by students and most (*n* = 136; 91%) reported at least one barrier to confronting these behaviours. Older students (OR = 0.89 [0.79 to 0.99]), individuals assigned female at birth (OR = 0.34 [0.15 to 0.77]), and students self-identifying as 2SLGBTQIA + (OR = 0.38 [0.15 to 0.94]) were less likely to believe their program was 2SLGBTQIA + inclusive. Older students (OR = 0.85 [0.76 to 0.94]) and 2SLGBTQIA + students (OR = 0.42 [0.23 to 0.76]) felt the same about their placements. Students who reported > 10 h of 2SLGBTQIA + training were more likely to believe their program was inclusive (OR = 3.18 [1.66 to 6.09]).

**Conclusions:**

Entry-level physiotherapy students in Canada show positive attitudes towards working with 2SLGBTQIA + persons but believe exposure to 2SLGBTQIA + health and inclusiveness is insufficient in their physiotherapy programs. This suggests greater attention dedicated to 2SLGBTQIA + health would be valued.

**Supplementary Information:**

The online version contains supplementary material available at 10.1186/s12889-023-16554-2.

## Background

The acronym 2SLGBTQIA + can be used to describe individuals who identify as Two-Spirit, lesbian, gay, bisexual, transgender, queer or questioning, intersex, asexual and additional identities not considered heterosexual (i.e., attracted to the opposite sex) or cisgender (i.e., identifying with a gender typically associated with their sex assigned at birth by societal standards). Individuals who identify as 2SLGBTQIA + experience worse health outcomes than heterosexual, cisgender peers [1–6] and face high rates of stigmatization and discrimination, social pressures/exclusion, trauma, abuse, poverty, and substance abuse [7–9]. Disparities also continue to widen as a sequela of the COVID-19 pandemic [10, 11].

Individuals of the 2SLGBTQIA + community lack safe, accessible healthcare environments, possibly due to hetero- and cis-normativity present in healthcare [12, 13]. Negative experiences with healthcare are documented by 2SLGBTQIA + individuals, including reports of discrimination and/or harassment or denial of care from health providers [14–17], leading many patients to delay medical care or forego it entirely [18–20]. In physiotherapy, patients who identify as 2SLGBTQIA + have reported incorrect assumptions about their sexuality or gender identity from their therapists, physical discomfort, fear of discrimination, and frustrations with educating their own healthcare professionals about 2SLGBTQIA + health needs [21]. Physiotherapists identifying as 2SLGBTQIA + have also highlighted the hetero- and cis-normative discourse present in physiotherapy practice, impacting 2SLGBTQIA + patients and peers alike [22].

Negative outcomes and experiences for 2SLGBTQIA + persons in healthcare may reflect the education and social environments provided in physiotherapy programs. Recent calls to action have emphasized the importance of 2SLGBTQIA + health and inclusiveness education for physiotherapy practice and advocate for its inclusion in entry-level programs [23, 24]. While 2SLGBTQIA + health education has been recently studied in physiotherapy programs internationally [25–27], it has yet to be evaluated in Canada, and no studies have evaluated 2SLGBTQIA + inclusiveness, specifically, in these programs.

As a physiotherapy profession, we must prioritize inclusive environments for individuals who identify as 2SLGBTQIA + to feel safe in our care. To understand how we can better meet the health needs of 2SLGBTQIA + populations and improve patient outcomes and experiences with healthcare, we must first understand the scope of 2SLGBTQIA + health education and inclusiveness provided in entry-level physiotherapy programs. The objectives of the study were to evaluate physiotherapy student attitudes, beliefs and perceptions related to 2SLGBTQIA + health education and working with individuals who identify as 2SLGBTQIA + in entry-level physiotherapy programs in Canada and evaluate physiotherapy program inclusiveness towards 2SLGBTQIA + persons.

## Methods

### Study design and recruitment

We completed a national, cross-sectional survey of students enrolled in entry-level physiotherapy programs across Canada between August and December 2021. In Canada, there are 15 physiotherapy programs, 10 in English and five in French, admitting approximately 1,200 students each year. Most Canadian physiotherapy programs last for two years and lead to a Master's degree. However, due to differences in provincial education structures, a few programs integrate Master's level training into undergraduate education, with programs lasting up to five years.

We included students who had completed a minimum of 6 months of their training through a Canadian physiotherapy program. This criterion was set to ensure respondents had acquired sufficient experience in the program to provide meaningful answers to the survey questions. In most programs, this corresponds to students in their second year of a 2-year degree, considering the survey was administered during the fall term when first-year students were commencing their program. We excluded any students who were < 6 months into their program or who were studying at an institution outside of Canada. Additionally, we excluded individuals who were not enrolled as physiotherapy students. We contacted chairpersons or program directors for each of the 15 Canadian entry-level physiotherapy programs to assist with the dissemination of a recruitment email to students in their physiotherapy program. We made two follow-up attempts with institutions from which we did not receive a response initially. Ultimately, we were able to establish contact with 10 out of the 15 Canadian institutions who agreed to share recruitment materials with their students. This included 1 out of 5 French institutions and 9 out of 10 English institutions. Recruitment materials were sent on behalf of program administrative staff from institutions who agreed to participate, which included a brief study description and a link to the survey. A reminder email was also sent to students one week following the initial email. A study recruitment flyer was also circulated through Twitter and Instagram (in both French and English, separately) by members of the research team to assist with recruitment. The survey was administered through Qualtrics XM, a software licensed by Western University, and survey responses were both anonymous and voluntary. Respondents were provided the option to complete the survey in French or English. Consistent with the study eligibility criteria, we asked if they were currently enrolled in a physiotherapy program at a Canadian university and had completed at least six months of the program before displaying the survey. If individuals responded "no", the survey ended. To ensure the survey content was applicable, clear, and unbiased [28], we ran a pre-test of the survey through individual one-on-one feedback sessions with four practicing physiotherapists (years since graduation were 1, 2, 5 and 9 years) and one physiotherapy student. These data were not used in the analyses presented. Two of these individuals were bilingual (French/English), one of which considered French as their first language. These individuals ensured the French translation of the full survey was accurate and appropriately reflected the content of the questions in English. The study was approved by Western University’s Research Ethics Board for Health Sciences Research Involving Human Subjects (REB # 119,132). All participants provided informed and written consent prior to participation in any study-related activities.

### Survey questions

The survey included questions related to demographics such as age, sex assigned at birth, gender identity, sexual orientation, race, ethnicity, religion, and university. Participants were also asked whether they identify as 2SLGBTQIA + . To evaluate student views on 2SLGBTQIA + health education and working with patients who identify as 2SLGBTQIA + , we asked participants about their attitudes and beliefs related to working with patients who identify as 2SLGBTQIA + , their perceptions on the level of 2SLGBTQIA + education in their program and whether this education should be mandatory. We also asked participants questions related to 2SLGBTQIA + inclusiveness in their physiotherapy program and on clinical placement and to report the number of hours they received related to 2SLGBTQIA + health in their program. Finally, we asked students about witnessing negative behaviours towards clients and/or peers who identify as 2SLGBTQIA + while in their program and/or on placement and any of their perceived barriers for addressing 2SLGBTQIA + discrimination in these settings. We based questions on content related to 2SLGBTQIPA + health education and inclusiveness and barriers identified through literature search and research team discussion.

Participants were asked to respond to questions in one of two ways. Questions were either provided on a 5-point Likert scale based on their level of agreement with a statement (*i.e., Strongly Disagree, Disagree, Neutral, Agree or Strongly Agree*) or on a scale of frequency (*i.e., Never, Rarely, Sometimes, Often or Always*) or were asked as *Yes*/*No* questions with an additional option for *Unsure*. For ease of interpretation, we collapsed similar responses from the 5-point Likert questions to a single measure (e.g., *Strongly Agree* and *Agree* became a single response of *Agree*). We therefore present the agreement data as *Agree, Neutral, or Disagree* and the frequency data as *Yes, Sometimes, or No*.

We also asked participants to complete The Lesbian, Gay, Bisexual, and Transgender Development of Clinical Skills Scale (LGBT-DOCSS) Questionnaire [29]. The LGBT-DOCSS has been shown to have test–retest reliability, internal consistency, and content/discriminant validity [29]. The LGBT-DOCSS is an 18-item interdisciplinary self-assessment for health-care providers to evaluate the clinical preparedness of clinicians for working with clients who identify as lesbian, gay, bisexual and/or trans. Items are 7-point Likert scales (1 = strongly disagree, 4 = somewhat agree/disagree, 7 = strongly agree). Summary scores are provided for 3 subscales (Clinical Preparedness [7 questions], Attitudinal Awareness [7 questions], and Basic Knowledge [4 questions]) that average selected items. There is also an overall mean score average of all items (Overall LGBT-DOCSS). A higher score on the LGBT-DOCSS (scored from 1 to 7) indicates higher levels of clinical preparedness and knowledge and less prejudice related to working with patients who identify as LGBT. The French version of the survey was translated by the first author (C.P.) whose first language is French, ensuring the translated questions accurately reflected the same content and framing of the English questions. These translations were also verified by two individuals during survey pre-testing.

### Analyses

We report demographic and survey questions as means with standard deviations or medians with interquartile range (IQR) for normally and non-normally distributed continuous data, respectively. We report frequencies with percentages for binary and ordinal outcomes. We also compared the four LGBT-DOCSS scores (clinical preparedness, attitudinal awareness, basic knowledge and overall) between individuals who identified as 2SLGBTQIA + and those who did not using independent T-tests, reported as mean differences (MD) with 95% confidence intervals (CI).

We also fitted various multivariable logistic regression analyses, reported as odds ratios (OR) with 95% CI. While running the analyses, we ensured the assumptions for logistic regression were met. We evaluated the association between potential predictors and three distinct outcomes: whether a student 1) felt their physiotherapy program did not provide sufficient knowledge about unique 2SLGBTQIA + healthcare needs, 2) believe their physiotherapy program is 2SLGBTQIA + -inclusive, and 3) believe their clinical placements are 2SLGBTQIA + -inclusive. We dichotomized outcomes based on students who responded *Yes* only (outcomes 2 and 3) or *No* only (outcome 1), depending on the way the question was framed for respondents in the survey. For all analyses, we selected potential predictor variables a priori which included age, sex assigned at birth, the number of hours of 2SLGBTQIA + training (i.e., less than 10 h or more than 10 h), and whether a student identifies as 2SLGBTQIA + (yes or no).

All quantitative data analyses were completed using Stata 16/IC (StataCorp LLC, College Station, TX) statistical software using a two-sided *p*-value < 0.05 to indicate statistical significance. We also report direct statements from students provided in an open textbox question which asked students to report any additional comments, questions, or concerns they wanted to share. We report open text responses for descriptive purposes. We did not perform a qualitative analysis.

## Results

A total 238 individuals engaged with the survey platform. Twenty-nine (12%) did not meet the inclusion criteria (i.e., were not physiotherapy students 6 + months into their program in Canada). Additionally, 45 respondents (19%) completed < 10% of the survey and were excluded, while 14 (6%) completed 10–50% and were also discarded. A total of 150 physiotherapy students in Canada, out of approximately 1,200 (i.e., 13%), completed the full survey with 35 students (23%) identifying as 2SLGBTQIA + and were included. From these respondents, 133 (89%) completed the survey in English, while 17 (11%) completed it in French. Table 1 presents a full summary of student respondent demographics. The median age was 24 (IQR, 23 to 26). Most students reported being assigned female at birth (*n* = 116, 77%), identified as heterosexual (*n* = 106, 71%) and cisgender (*n* = 144, 96%), were white (*n* = 121, 81%) and self-identified Canadian (*n* = 121, 81%) as their ethnicity. Various religions were represented in the sample, however no religion (*n* = 62, 41%) and Christian (*n* = 44, 29%) were most frequently reported. Students from 12 different Canadian universities completed the survey with representation from Alberta, British Columbia, Manitoba, Nova Scotia, Ontario, Québec, and Saskatchewan.

**Table 1:** Baseline demographics and clinical characteristics

Demographic/clinical characteristic	Total (*n* = 150)
**Age**, years (median, IQR)	24 (23 to 26)
**Sex assigned at birth,** no. (%)	
Male	34 (23%)
Female	116 (77%)
Intersex	0 (0%)
Preferred not to disclose	0 (0%)
**Sexual orientation,** no. (%)	
Asexual	3 (2%)
Bisexual	22 (15%)
Gay	4 (3%)
Heterosexual (straight)	106 (71%)
Lesbian	11 (7%)
Pansexual	3 (2%)
Panromantic	1 (1%)
Queer	8 (5%)
Questioning	10 (7%)
Preferred not to disclose	1 (1%)
**Gender identity,** no. (%)	
Man, or primarily masculine	35 (23%)
Woman, or primarily feminine	109 (73%)
Indigenous or other cultural gender minority (e.g., Two-Spirit)	0 (0%)
Neither man, nor woman (e.g., gender diverse, gender fluid, non-binary, agender)	6 (4%)
Transgender man	0 (0%)
Transgender woman	0 (0%)
Preferred not to disclose	0 (0%)
**Race,** no. (%)	
Arab	4 (3%)
Black	2 (1%)
Chinese	13 (9%)
Filipino	1 (1%)
Japanese	0 (0%)
Jewish	1 (1%)
Korean	0 (0%)
Latin American	2 (1%)
South Asian (e.g., East Indian, Pakistani, Sri Lankan, etc.)	6 (4%)
Southeast Asian (e.g., Vietnamese, Cambodian, Thai, etc.)	2 (1%)
West Asian	0 (0%)
White	121 (81%)
Mixed race	8 (5%)
Preferred not to disclose	0 (0%)
**Ethnicity,** no. (%)	
African – Central or West (including, but not limited to Liberian, Nigerian, Senegalese)	0 (0%)
African – Northern (*including, but not limited to* Egyptian, Libyan, Tunisian)	1 (1%)
African – Southern or Eastern (*including, but not limited to* Kenyan, South African, Ugandan)	1 (1%)
American	1 (1%)
Asian – South (*including, but not limited to* Punjabi, Sri Lankan, Tamil)	9 (6%)
Asian – East or Southeast (*including, but not limited to* Burmese, Filipino, Hmong, Indonesian, Laotian, Malaysian, Mien, Singaporean, Thai, Vietnamese)	13 (9%)
Canadian	121 (81%)
Caribbean (*including, but not limited to* Afro-Caribbean, Asian-Caribbean, Latinx-Caribbean, Indo-Caribbean)	4 (3%)
European – British (*eg,* English, Irish, Scottish)	33 (22%)
European – French (*eg,* Breton, French)	4 (3%)
European – Western (*including, but not limited to* Austrian, German, Slovenian)	13 (9%)
European – Northern (*including, but not limited to* Danish, Finnish, Swedish)	2 (1%)
European – Eastern (*including, but not limited to* Hungarian, Polish, Ukrainian)	13 (9%)
European – Southern (*including, but not limited to* Greek, Italian, Spanish)	13 (9%)
Indigenous (First Nations, Inuit, Métis, Native American)	3 (2%)
Latin, Central and South American (*including, but not limited to* Brazilian, Chilean, Mexican)	0 (0%)
Middle Eastern (e.g., Afghan, Iranian, Iraqi, Israeli, Lebanese)	10 (7%)
Oceania (Australian and New Zealand)	0 (0%)
Pacific Islands (Fijian, Hawaiian, Samoan)	0 (0%)
Québécois(e)	3 (2%)
Preferred not to disclose	0 (0%)
**Religion**, no. (%)	
No religion	62 (41%)
Agnostic	17 (11%)
Atheist	8 (5%)
Buddhist	1 (1%)
Christian	44 (29%)
Muslim	7 (5%)
Jewish	6 (4%)
Hellenistic	0 (0%)
Hindu	2 (1%)
Traditional or folk religion, Folk religion, Spiritist	3 (2%)
Preferred not to disclose	2 (1%)
**University**, no. (%)	
Dalhousie University	7 (5%)
McGill University	14 (9%)
McMaster University	13 (9%)
Queen’s University	8 (5%)
Université de Montréal	0 (0%)
Université de Sherbrooke	0 (0%)
Université du Québec à Chicoutimi	0 (0%)
Université d’Ottawa	2 (1%)
Université Laval	14 (9%)
University of Alberta	5 (3%)
University of British Columbia	16 (11%)
University of Manitoba	6 (4%)
University of Saskatchewan	7 (5%)
University of Toronto	8 (5%)
Western University	50 (33%)

### Attitudes and beliefs

**Fig. 1:**
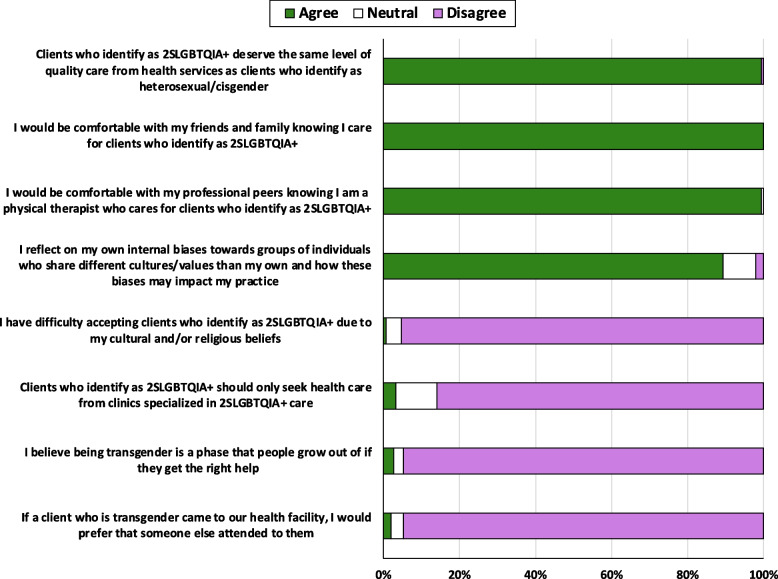
Self-reported physiotherapy student (*n* = 150) attitudes and beliefs related to working with clients who identify as 2SLGBTQIA + in a physiotherapy setting, reported as percentage of students

Nearly all students agreed clients who identify as 2SLGBTQIA + deserve the same quality of care as clients who identify as heterosexual and cisgender (*n* = 149, 99%), and were comfortable with friends and family (*n* = 150, 100%) and professional peers (*n* = 149, 99%) knowing they care for 2SLGBTQIA + clients (Fig. 1). Many students reflected on internal biases related to working with individuals who share different cultures/values than their own (*n* = 134, 89%). Most students disagreed with the statements of having difficulty accepting 2SLGBTQIA + clients due to cultural/religious beliefs (*n* = 143, 95%) or that clients who identify as 2SLGBTQIA + should seek care from specialized clinics (*n* = 129, 86%). They also largely disagreed in believing a transgender client will “grow out of it” if they get the right help (*n* = 142, 95%) or preferring another clinician attend to a transgender client who came into their facility (*n* = 142, 95%) (Fig. 1).

### Perceptions

**Fig. 2:**
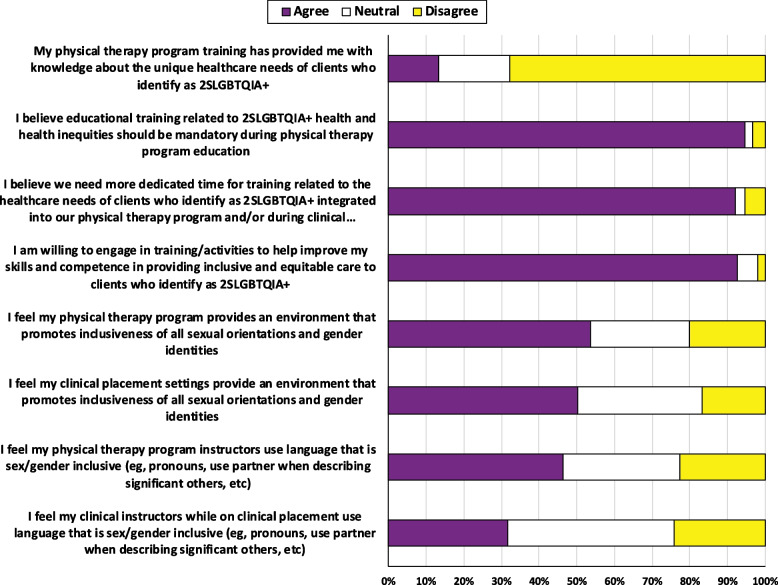
Self-reported physiotherapy student (*n* = 150) perceptions related to their physiotherapy education for working clients who identify as 2SLGBTQIA + in a physiotherapy setting, reported as percentage of students

Few students (*n* = 20; 13%) believed their physiotherapy program training provided them with knowledge about the unique healthcare needs of clients who identify as 2SLGBTQIA + (Fig. 2). Forty-four respondents (29%) reported zero training hours dedicated to 2SLGBTQIA + health education while in school, whereas 70 (47%) reported between 0–10 h and 36 (24%) reported > 10 h. On the other hand, most respondents believed educational training related to 2SLGBTQIA + health should be mandatory in their physiotherapy programs (*n* = 141, 94%), that more time should be dedicated to 2SLGBTQIA + health education in the physiotherapy curriculum (*n* = 137, 92%) and were willing to engage in additional training to help improve their skills in working with 2SLGBTQIA + persons (*n* = 138, 92%).

### Program inclusiveness

Approximately half of students believed their training program (*n* = 80, 54%) and clinical placements (*n* = 75, 50%) promoted inclusiveness for 2SLGBTQIA + individuals (Fig. 2). Physiotherapy program instructors and clinical placement supervisors were believed to use inclusive sex/gender language by 69 (46%) and 47 (31%) of students, respectively (Fig. 2). There was also a lot of uncertainty (26–44% neutral responses) for these four questions.

**Fig. 3:**
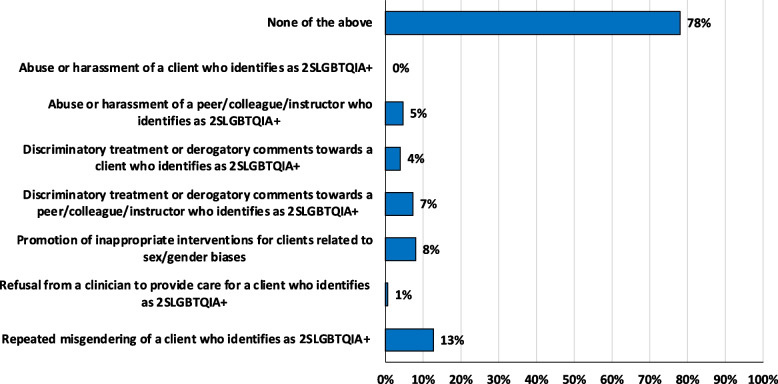
Self-reported physiotherapy student (*n* = 150) witnessed behaviours towards clients and/or peers who identify as 2SLGBTQIA + while in their physiotherapy program and/or while on clinical placement, reported as percentage of students

Although many students (*n* = 117, 78%) did not witness negative behaviours towards clients and/or peers who identify as 2SLGBTQIA + while in their physiotherapy program or on clinical placement, a total of 56 acts of discrimination, harassment and/or abuse towards 2SLGBTQIA + clients and/or peers were reported by students (Fig. 3).

**Fig. 4:**
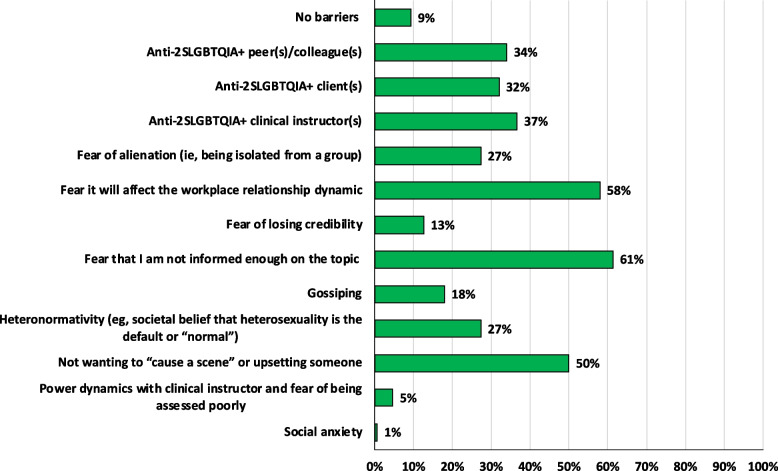
Self-reported physiotherapy student (*n* = 150) barriers to confronting a peer or colleague who is discriminatory towards an individual who identifies as 2SLGBTQIA + while in their physiotherapy program and/or while on clinical placement, reported as percentage of students

Most students reported barriers for confronting a peer or colleague who is discriminatory towards an individual who identifies as 2SLGBTQIA + . Specifically, fear of not being well-informed (*n* = 92, 61%), apprehension of affecting the workplace dynamic (*n* = 87, 58%) and not wanting to “cause a scene” or upset someone (*n* = 75, 50%) were the most frequently reported barriers. Additional barriers included the presence of anti-2SLGBTQIA + peers/colleagues (*n* = 51, 34%), clients (*n* = 48, 32%), and clinical instructors (*n* = 55, 37%), and fear of alienation (*n* = 41, 27%). A few students reported no barriers (*n* = 14, 9%). All barriers are reported in Fig. 4.

### LGBT-DOCSS scores

Group mean scores for the LGBT-DOCSS subscales were 3.79 ± 1.02 points for clinical preparedness, 6.73 ± 0.67 points for attitudes, and 5.13 ± 1.14 points for knowledge, and the overall mean score was 5.10 ± 0.66 points. On average, students who identified as 2SLGBTQIA + scored better than those who did not for the overall LGBT-DOCSS score (MD = 0.46 [95%CI, 0.21 to 0.70]), clinical preparedness (MD = 0.51 [95%CI, 0.12 to 0.89]), and attitudinal awareness (MD = 0.29 [95%CI, 0.04 to 0.54]). The difference in basic knowledge was not significant (MD = 0.38 [95%CI, -0.05 to 0.82]). Graphical representation of group comparisons is also provided in Supplemental Figure 1.

### Logistic regression

For every one additional year of age, students were at reduced odds of believing their physiotherapy program (OR = 0.89 [95%CI, 0.79 to 0.99], *p* = 0.45) and clinical placements (OR = 0.85 [95%CI, 0.76 to 0.94], *p* < 0.01) were 2SLGBTQIA + -inclusive (Table 2). Students assigned female at birth were at increased odds (OR = 3.15 [95%CI, 1.71 to 5.82], *p* < 0.01) of feeling their physiotherapy program did not provide sufficient knowledge about unique 2SLGBTQIA + healthcare needs and reduced odds (OR = 0.34 [95%CI, 0.15 to 0.77], *p* = 0.01) of believing their physiotherapy program was 2SLGBTQIA + -inclusive when compared to students assigned male at birth. Students who received 10 or more hours of 2SLGBTQIA + health education training were at increased odds (OR = 3.18 [95%CI, 1.66 to 6.09], *p* < 0.01) of believing their physiotherapy program was 2SLGBTQIA + -inclusive compared to students who received < 10 h. Identifying as 2SLGBTQIA + reduced the odds of students believing their physiotherapy program (OR = 0.38 [95%CI, 0.15 to 0.94], *p* = 0.03) and clinical placements (OR = 0.42 [95%CI, 0.23 to 0.76], *p* < 0.01) were 2SLGBTQIA + -inclusive.

**Table 2:** Results of the logistic regression models (*n* = 150)

Predictors	Odds Ratio (95%CIs)		
	Felt their PT program did not provide sufficient knowledge about unique 2SLGBTQIA + healthcare needs	Believe their physiotherapy program is 2SLGBTQIA + -inclusive	Believe their clinical placements are 2SLGBTQIA + -inclusive
**Age**	1.08 (0.91 to 1.29)	**0.89 (0.79 to 0.99)**	**0.85 (0.76 to 0.94)**
**Sex assigned at birth**^a^			
Male	Ref	Ref	Ref
Female	**3.15 (1.71 to 5.82)**	**0.34 (0.15 to 0.77)**	0.52 (0.17 to 1.53)
**10 or more hours of 2SLGBTQIA + training**			
< 10 h	Ref	Ref	Ref
10 + hours	0.28 (0.08 to 1.01)	**3.18 (1.66 to 6.09)**	1.69 (0.72 to 3.95)
**Identifies as 2SLGBTQIA + **			
No	Ref	Ref	Ref
Yes	1.03 (0.48 to 2.21)	**0.38 (0.15 to 0.94)**	**0.42 (0.23 to 0.76)**

**Bolded** estimates represent statistically significant associations at the 5% level

The variance was adjusted for university attended using robust sandwich estimators

*Abbreviations*: *2SLGBTQIA* + Two-spirit, lesbian, gay, bisexual, transgender, queer/questioning, intersex, asexual/aromantic and all additional identities not considered heterosexual and cisgender, *CI* Confidence intervals, *PT* Physiotherapy, *Ref.* Reference variable

^a^Intersex was also included as a survey response option, however no students reported intersex as their sex assigned at birth

### Open-text responses

Direct statements from students for a question asking them to report any additional comments, questions, or concerns they wanted to share are provided in Table 3. Of note, the results do not include responses from students who identify as 2SLGBTQIA + . We report open text responses for descriptive purposes. We did not perform a qualitative analysis.

**Table 3:** Direct statements from students

Students were asked: *If you have any additional comments, questions, or concerns you would like to share, please provide them in the text box below*	
*“More training and learning of the inequities faced would be helpful in understanding how to make a change in our profession.”*	*“I would love more education in PT school surrounding 2SLGBTQIA* + *patients.”*
*“Thank you for initiating this research, this is an important topic that does not receive enough attention”*	*“Thank you for doing this research, it is changing/benefitting the lives of many.”*
*“It would be amazing to see more education and resources about 2SLGBTQIA* + *integrated in the MPT curriculum.”*	*“All of the clinical skills are demonstrated on heteronormative cisgender men at [university]”*
^a^* “I find that the issues of health and access for these clienteles are relevant to explore in a course, but acceptance and openness to others, as well as knowledge of the plurality of identities should be basic knowledge and innate. I would be interested in a course that talks about clinical conduct with this clientele, but not a course that teaches me who this clientele is. We are in 2021, we should know.”*	*“As a heterosexual, cisgender female, more education (and practical scenarios) on how to treat individuals and things to assess differently in transgender clients/patients related to their specific health requirements (e.g., bottom/top surgeries, binding, *etc*.) would be useful. I am also curious on how to conduct treatments/assessments with LGB individuals that may be different from hetero individuals, if any.”*
*“My program has discussed gender identity and associated health inequalities and gender-affirming procedures more than the inequalities that LGB individuals face. We have not talked at all about two-spirit individuals. Despite your introduction regarding intersectionality, with all the inequities that Indigenous people face, and how prevalent those issues have become in the media recently, I would have been interested in answering more questions directly related to the two-spirit community. My program did not touch on intersectionality. With our lessons related to gender, as well as other lessons related specifically to Indigenous culture and health, I feel that this was a missed opportunity to learn about intersectionality. I am aware that my program and university highlight Indigenous issues (more so than other schools in my personal experience and through conversations with peers). If this is the case, and even we aren’t learning about two-spirited individuals, who is? Again, it seems that the Physiotherapy education in [province] is missing this relevant and important topic.”*	*“We do have sessions on the basics of queer identity, but we need a lot more as it relates to treatment especially for transgender folks. For example, a lot of cardiac or respiratory equations use sex as a variable. Further, we need training on how to develop safe language with a patient on how to refer to their body or mobilize/touch their body. Also, we could use dos and don’ts, such as not telling a trans patient you couldn’t tell they were trans. Overall we need more time for discussion. We had an anonymous whiteboard for our session and one person kept repeatedly posting “why can’t people just choose one gender” when introduced to the concept of gender fluid. It was unclear why, but their question was not addressed.”*

^a^Statement has been translated from French to English by author C.P., a fluently bilingual researcher, who attests the translated materials accurately reflect the message from its original French version

## Discussion

This is the first study to evaluate physiotherapy student attitudes, beliefs and perceptions related to 2SLGBTQIA + health education and working with individuals who identify as 2SLGBTQIA + in entry-level physiotherapy programs in Canada. It is also the first to explore 2SLGBTQIA + inclusiveness in entry-level physiotherapy programs.

### Attitudes and beliefs

Our findings suggest physiotherapy students in Canada show positive attitudes towards working with individuals who identify as 2SLGBTQIA + which is consistent with recent studies completed internationally [25, 26]. They are particularly encouraging as a previous study reported a lack of support from physiotherapists in working with clients who identify as 2SLGBTQIA +  [30]. Overall, these results suggest views of 2SLGBTQIA + acceptance in physiotherapy has improved [31] and a generational shift in beliefs may be on the horizon for the physiotherapy profession. These positive attitudes may be related to the introduction of anti-discrimination laws in Canada, including same-sex marriage legalization in 2005 and protection against gender identity/expression discrimination by Canadian Human Rights Act and Criminal Code in 2017. Similarly, Canada was the first country to provide Census data (2021) on non-binary and transgender individuals [32]. However, despite these important steps towards greater inclusion in Canada, the 2SLGBTQIA + community continues to face substantial barriers accessing safe and inclusive spaces for healthcare [12, 13] and is met with various forms of discrimination in healthcare settings [14–17], including in physiotherapy [21]. As the new generations begins their careers, we may see changes in these behaviours and experiences, but results from the present study show greater attention to 2SLGBTQIA + inclusiveness in entry-level programs is warranted.

Poor experiences within physiotherapy for 2SLGBTQIA + persons may not be driven by negative views from therapists towards 2SLGBTQIA + persons but other sources, such as a lack of awareness for the relevance of sex/gender identity in physiotherapy practice. Some students may hold the view “treating everyone the same” will lead to equity in practice, which may further perpetuate health inequities by glossing over the processes of stigmatization and discrimination faced by members of the 2SLGBTQIA + community. Therefore, students may not be adequately equipped with the necessary knowledge to provide equitable patient-centered care with a lens of cultural humility [33]. Some students may also believe 2SLGBTQIA + health education should be considered a “special topic” for continuing education. However, inclusive care for 2SLGBTQIA + persons should be fundamental for all practicing physiotherapists as physiotherapists interact with 2SLGBTQIA + persons daily. Further work to examine these concepts is warranted. Ultimately, the evidence shows physiotherapists lack knowledge regarding inclusive practice and 2SLGBTQIA + health which is an ongoing area of frustration for both 2SLGBTQIA + patients and clinicians [21, 22].

### Perceptions

Few students (13%) believed their entry-level physiotherapy program provided them with sufficient knowledge about the unique healthcare needs of clients who identify as 2SLGBTQIA + . While previous studies suggest a minimum of 35 h of 2LGBTQIA + training in health programs is necessary to achieve cultural competency [34], only 4 students (3%) from the present study reported 35 + hours of 2SLGBTQIA + education in their physiotherapy program, while 29% reported none. These results are analogous to previous studies reporting similar results in the UK [25] and US [26]. There is a need for greater emphasis on 2SLGBTQIA + health education not only in Canada but also internationally.

Currently, no specific standards have been outlined by the Canadian Council of Physiotherapy University Programs (CCPUP) for incorporating 2SLGBTQIA + health education and competency objectives in the Canadian physiotherapy curriculum [24, 35]. Perhaps the first steps to improving 2SLGBTQIA + education and inclusivity in physiotherapy programs are to develop a set of competencies related to sex and gender diversity in the curriculum and evaluate how effectively they are implemented at the institutional level. For example, one student reported through an open-text response that while concepts related to sex and gender were implemented in their program, student queries were not being adequately addressed by course instructors, suggesting there may also be a need for greater 2SLGBTQIA + training for educators teaching in entry-level physiotherapy programs. Importantly, we acknowledge the statement was provided through an open text response and was not evaluated using rigorous qualitative methods. Nonetheless, next steps in this area of research could include exploring educator knowledge and preparedness and questions related to strategies for effective implementation of competencies. For example, physiotherapy programs could use the tool for assessing LGBTQI + health training (TAHLT) [36] as a guide to assess and improve their 2SLGBTQIA + curriculum content, and to help train faculty on incorporating 2SLGBTQIA + care into teaching.

Students showed positive attitudes towards inclusion of more health education and inclusivity training related to working with individuals who identify as 2SLGBTQIA + in physiotherapy entry-level programs. Students noted there is a need to dedicate more time to 2SLGBTQIA + education in entry-level physiotherapy programs (92%), with most agreeing it should be a mandatory (94%) and willing to engage in more learning to improve their 2SLGBTQIA + knowledge (92%). Similar findings have been reported previously where students did not feel prepared to address 2SLGBTQIA + health concerns but expressed interest in receiving additional education in their physiotherapy program [26]. When provided the opportunity to share open text responses, nine students also emphasized wanting to learn more about 2SLBGTQIA + health in their physiotherapy programs and believe the topic currently does not receive enough attention in entry-level physiotherapy programs.

### Program inclusiveness

Around half of student respondents believed their program or clinical placements promoted inclusiveness but fewer reported their program instructors and clinical instructors used sex and gender inclusive language. The overall uncertainty in responding to questions about inclusiveness suggests there may be a lack of awareness and/or confusion surrounding what constitutes an inclusive environment for individuals who identify as 2SLGBTQIA + .

Interestingly, older students and students assigned female at birth were less likely to believe their physiotherapy education was inclusive. Individuals assigned female at birth were also less likely to feel their programs provided them with sufficient knowledge about 2SLGBTQIA + health needs. These results are not surprising as females have also reported experiencing discrimination and harassment while in healthcare settings [37] and it may therefore help facilitate empathy for 2SLGBTQIA + persons because of previous personal experiences. Moreover, we found a greater number of training hours was associated with increased odds of students believing their program is more 2SLGBTQIA + inclusive. Curriculums that include more 2SLGBTQIA + content may foster a more welcoming learning environment for 2SLGBTQIA + physiotherapy students, however greater attention and prioritization must also be placed on inclusiveness by physiotherapy programs.

We believe this is the first study to report behaviours of discrimination and/or abuse towards clients and/or peers who identify as 2SLGBTQIA + observed by physiotherapy students while in their program and/or on clinical placement. Although 78% of students did not report any such behaviours, 56 acts of discrimination, harassment and/or abuse were reported by students, including repeated misgendering, promotion of inappropriate interventions for clients related to sex/gender biases, client discrimination, peer discrimination, and peer abuse and/or harassment regarding their 2SLGBTQIA + identities. Students also reported a variety of barriers to confronting a peer or colleague who is discriminatory towards a 2SLGBTQIA + individual which suggests these behaviours may remain unchallenged and, thus, inherently be deemed tolerable in these learning environments. While some individuals may argue sex and gender is not a relevant topic to physiotherapy practice, our results suggest the opposite. It is important for entry-level programs to prioritize inclusive environments for everyone, so students, peers and patients alike feel safe. Practicing effective critical allyship [38] and being active bystanders when witnessing discriminatory behaviours [39] should be priorities for physiotherapy programs.

Importantly, when students were asked about barriers to confronting a peer or colleague who is discriminatory towards an individual who identifies as 2SLGBTQIA + , 61% expressed fear of not being well-informed. These results further suggest greater 2SLGBTQIA + education may also help create more supportive and inclusive environments for 2SLGBTQIA + persons. Perceived barriers related to social environment were also expressed including apprehension of affecting the workplace dynamic, not wanting to “cause a scene” or upset someone, anti-2SLGBTQIA + peers/colleagues, clients, and clinical instructors, and fear of alienation. These findings underscore the importance of assessing the clinical environment's inclusiveness towards 2SLGBTQIA + individuals among licensed physiotherapists practicing in Canada. The findings also highlight the lack of inclusivity in learning environments for physiotherapy students in Canada.

Potential reasons for perceived lack of inclusivity in programs include a lack of 2SLGBTQIA + representation among staff, hesitancy or a lack of expertise from staff to incorporate 2SLGBTQIA + content in teaching, difficulty incorporating content due to program constraints, beliefs that 2SLGBTQIA + status is irrelevant to physiotherapy, and/or anti-2SLGBTQIA + staff. These questions should be explored in subsequent studies, and we should also explore qualitative studies with 2SLGBTQIA + students to better understand their experiences while in their physiotherapy program and their perspectives on areas for improvement.

### LGBT-DOCSS

Scores from the LGBT-DOCSS suggest students generally had positive attitudes towards working with 2SLGBTQIA + patients and showed a moderate degree of knowledge about 2SLGBTQIA + considerations, However, students showed a lack of clinical preparedness in working with the 2SLGBTQIA + population. The findings are similar to previous studies that used LGBT-DOCSS to evaluate competencies in medical [34, 40, 41] and pharmacy [42] students, and with students from a variety of health disciplines [43]. These results emphasize the importance of 2SLGBTQIA + education in physiotherapy programs and suggest incorporating educational interventions focused on developing practical skills and competencies (e.g., practical simulations, case discussions, interviews) could be particularly beneficial [44].

### 2SLGBTQIA + Identity

Our study results also highlight differences in survey responses between students who identify as 2SLGBTQIA + and heterosexual, cisgender students, which is similar to findings reported in the UK [25] and US [26]. We found students who identify as 2SLGBTQIA + showed greater clinical preparedness and attitudinal awareness in working with patients who identify as 2SLGBTQIA + based on their LGBT-DOCSS scores. Students were also more likely to feel confident in their ability to communicate with 2SLGBTQIA + clients and were less likely to believe their physiotherapy program and clinical placements are inclusive to 2SLGBTQIA + persons. These results are not surprising as 2SLGBTQIA + students may have more experience communicating on a regular basis with individuals who share similar identities, and thus may help with building connection with patients who identify as 2SLGBTQIA + . Additionally, 2SLGBTQIA + students are also likely to have a greater understanding and heightened awareness of 2SLGBTQIA + inclusivity based on personal and lived experiences and, subsequently, have a better knowledge of potential 2SLGBTQIA + microaggressions that could threaten 2SLGBTQIA + person safety and/or feelings of inclusion [38]. However, it is important to highlight again these sentiments were also expressed by heterosexual, cisgender students, and explanations and potential strategies for improving physiotherapy program inclusivity will need to be explored in future studies.

### Limitations and generalizability

The 150 students who completed the survey only represent approximately 13% of current physiotherapy students in Canada and fewer responses were received from central (e.g., Manitoba, Saskatchewan, and Alberta) and eastern (e.g., Dalhousie) Canadian provinces. Therefore, our results may not be generalizable to all physiotherapy students in Canada. Furthermore, while French programs make up around 30% of physiotherapy students in Canada, our survey responses from French programs comprised only 10% of the total. This discrepancy is expected considering only one out of the five French institutions we contacted agreed to share recruitment materials for our survey with their students. We also acknowledge that we assume respondents answered questions honestly and completed the survey only once. While we asked potential participants to only complete the survey once in the letter of information and consent, due to the anonymity of the survey, we are unable to confirm whether respondents completed the survey more than once. The French translation of the LGBT-DOCSS questionnaire has also not been validated. However, it was translated by the first author (C.P.), a francophone, and verified by two francophones during survey pre-testing. Students who had more vested interest in the research topic (positively or negatively) may have been more willing to complete the survey. That is, 23% of students identified as 2SLGBTQIA + which is higher than the national proportion of reported individuals who self-identify as 2SLGBTQIA + in Canada (4%) [8], suggesting possible self-selection bias [45]. Most respondents also identified as women and assigned female at birth, which may impact generalizability of results. Alternatively, some questions from the survey could be triggering for some students and may have deterred them from completing the survey. However, we attempted to account for this by anonymizing the survey to protect confidentiality [46], and this was outlined for participants in the letter of information and consent prior to participation. Finally, Hawthorne effects may have led students to feel the need to answer questions a specific way from pressures of expected answers from society and may therefore not reflect their true perceptions [47]. Anonymization of the survey results should also address this.

## Conclusion

While physiotherapy students in Canada show positive attitudes towards working with individuals who identify as 2SLGBTQIA + , they consider their exposure to 2SLGBTQIA + health as insufficient in their entry-level physiotherapy programs. Students believe greater attention towards 2SLGBTQIA + education is needed and show a strong willingness to learn. There also appears to be a lack of 2SLGBTQIA + inclusiveness in Canadian physiotherapy programs, including reports of witnessing 2SLGBTQIA + discrimination in their program or on placement. These findings suggest greater attention to 2SLGBTQIA + health and inclusion training in Canadian physiotherapy programs would be greatly valued and is needed.

### Supplementary Information

**Additional file 1: Appendix A.** Supplemental material. **Supplemental Figure 1.** Canadian physiotherapy students (*n*=150) group means for students who identify as cisgender and heterosexual (*n*=115) and for students who identify as 2SLGBTQIA+ (*n*=35) for the Overall, Clinical Preparedness, Attitudinal Awareness, and Basic Knowledge subscales of the Lesbian, Gay, Bisexual, and Transgender Development of Clinical Skills Scale (LGBT-DOCSS) Questionnaire. Each subscale is scored from 0 to 7 points.

## Data Availability

The datasets generated during and/or analysed during the current study are available from the corresponding author on reasonable request.

## Abbreviations

2SLGBTQIA +: Individuals identifying as Two-Spirit, lesbian, gay, bisexual, transgender, queer or questioning, intersex, asexual and additional sexual orientations, and gender identities not considered heterosexual and/or cisgender
CCPUP: Canadian Council of Physiotherapy University Programs
CI: Confidence Interval
COVID-19: Severe acute respiratory syndrome coronavirus 2 (SARS-CoV-2)
IQR: Interquartile Range
LGBT-DOCSS: The Lesbian, Gay, Bisexual, and Transgender Development of Clinical Skills Scale Questionnaire
MD: Mean difference
OR: Odds Ratio
REB: Research Ethics Board
