# Supplementary material for: Students’ attitudes, beliefs and perceptions surrounding 2SLGBTQIA + health education and inclusiveness in Canadian physiotherapy programs
Source: BMC Public Health. 2023 Aug 30;23:1661. doi: 10.1186/s12889-023-16554-2 (PMC10466852; doi:10.1186/s12889-023-16554-2)
Supplement: Supplementary file 1 — Additional file 1: Appendix A. Supplemental material. Supplemental Figure 1. Canadian physiotherapy students (n=150) group means for students who identify as cisgender and heterosexual (n=115) and for students who identify as 2SLGBTQIA+ (n=35) for the Overall, Clinical Preparedness, Attitudinal Awareness, and Basic Knowledge subscales of the Lesbian, Gay, Bisexual, and Transgender Development of Clinical Skills Scale (LGBT-DOCSS) Questionnaire. Each subscale is scored from 0 to 7 points. [file 12889_2023_16554_MOESM1_ESM.docx]

**APPENDIX A: SUPPLEMENTAL MATERIAL**

**Supplemental Figure 1. Canadian physiotherapy students (n=150) group means for students who identify as cisgender and heterosexual (n=115) and for students who identify as 2SLGBTQIA+ (n=35) for the Overall, Clinical Preparedness, Attitudinal Awareness, and Basic Knowledge subscales of the Lesbian, Gay, Bisexual, and Transgender Development of Clinical Skills Scale (LGBT-DOCSS) Questionnaire. Each subscale is scored from 0 to 7 points.**
